# Initiation of combined antiretroviral therapy confers suboptimal beneficial effects on neurovascular function in people with HIV

**DOI:** 10.3389/fneur.2023.1240300

**Published:** 2023-08-31

**Authors:** Meera V. Singh, Md Nasir Uddin, Vir B. Singh, Angelique N. Peterson, Kyle D. Murray, Yuchuan Zhuang, Alicia Tyrell, Lu Wang, Madalina E. Tivarus, Jianhui Zhong, Xing Qiu, Giovanni Schifitto

**Affiliations:** ^1^Department of Neurology, University of Rochester, Rochester, NY, United States; ^2^Department of Microbiology and Immunology, University of Rochester, Rochester, NY, United States; ^3^Department of Biomedical Engineering, University of Rochester, Rochester, NY, United States; ^4^Albany College of Pharmacy and Health Sciences, Albany, NY, United States; ^5^Department of Physics and Astronomy, University of Rochester, Rochester, NY, United States; ^6^Department of Electrical and Computer Engineering, University of Rochester, Rochester, NY, United States; ^7^Clinical and Translational Science Institute, University of Rochester, Rochester, NY, United States; ^8^Department of Biostatistics and Computational Biology, University of Rochester, Rochester, NY, United States; ^9^Department of Imaging Sciences, University of Rochester, Rochester, NY, United States; ^10^Department of Neuroscience, University of Rochester, Rochester, NY, United States

**Keywords:** HIV, cART, neuroinflammation, neuroimaging, cerebral vascular reactivity, cerebral blood flow, resting-state fMRI, microparticles

## Abstract

**Introduction:**

Due to advances in combined anti-retroviral treatment (cART), there is an increased burden of age-related cerebrovascular disease (CBVD), in people living with HIV (PWH). The underlying CNS injury can be assessed by measuring cerebral blood flow (CBF) and cerebrovascular reactivity (CVR).

**Methods:**

35 treatment-naïve PWH and 53 HIV negative controls (HC) were enrolled in this study. Study participants underwent T1-weighted anatomical, pseudo-continuous arterial spin labeling, and resting-state functional MRI to obtain measures of CBF and CVR prior to starting cART treatment and at two-time points (12 weeks and 2 years) post-cART initiation. Controls were scanned at the baseline and 2-year visits. We also measured plasma levels of microparticles of endothelial and glial origin and well-known endothelial inflammation markers, ICAM-1 and VCAM-1, to assess HIV-associated endothelial inflammation and the interaction of these peripheral markers with brain neurovascular function.

**Results:**

HIV infection was found to be associated with reduced CVR and increased levels of endothelial and glial microparticles (MPs) prior to initiation of cART. Further, CVR correlated negatively with peripheral MP levels in PWH.

**Discussion:**

Our results suggest that while cART treatment has a beneficial effect on the neurovascular function after initiation, these benefits are suboptimal over time.

## 1. Introduction

Combination antiretroviral therapy (cART) has dramatically changed the outlook of infection with Human Immunodeficiency Virus, type 1 (HIV), from one with no treatment to that of a manageable chronic disease, and people with HIV (PWH) now have a lifespan getting closer to those uninfected albeit with a larger burden of comorbidities, including vascular disease (1). Cerebrovascular disease (CBVD) is associated with aging and is more frequent among PWH (2). It poses a significant disease burden in this vulnerable population, especially as nearly half of PWH in USA are age 50 and older. CBVD is a leading cause of vascular cognitive impairment and dementia that may further contribute to HIV-associated cognitive impairment (3–7).

HIV is a neurotropic virus and is known to enter the central nervous system (CNS) as early as 8 days post infection (8). Of interest, HIV can also infect pericytes, a key cellular component of the blood–brain barrier (BBB) (9). Systemic chronic inflammation associated with HIV infection may also contribute to BBB permeability (10). Similarly, cART effect on endothelial cell may facilitate BBB disruption (11). The aging HIV population also faces traditional risk factors, including diabetes, dyslipidemia, and cardiovascular disease, that can contribute to CBVD. Large epidemiological studies suggest that CBVD is more common in PWH compared to HIV uninfected individuals and is considered a major driver of CNS injury (3–7).

Advanced neuroimaging techniques such as arterial spin labeling (ASL) and resting-state functional magnetic resonance imaging (rs-fMRI) allow non-invasive assessment of cerebral blood flow (CBF) and cerebrovascular reactivity (CVR), respectively (12–17). CBF is a measure of brain perfusion and can be used to assess both local changes in perfusion pressure and redistribution of blood flow within that region’s vascular network. CBF is tightly coupled to neuronal function, though several components of the functional neurovascular unit (neurons, astrocytes, endothelial, and pericytes) contribute to overall CBF regulation (18). CVR reflects a compensatory change in cerebral arteries in response to a vasoactive stimulus such as CO_2_ to meet the changing blood supply demands of the CNS (13). Since decreased CVR is associated with CBVD, CVR mapping can be used as an imaging marker to assess cerebral vascular territories with or at risk of CBVD (19). There are a few reports that have investigated CBF in PWH. Ances et al. (20) have shown reduced resting CBF in the basal ganglia and visual cortex of PWH compared to HIV negative healthy controls (HC). CBF abnormalities have also been documented in PWH with cognitive impairment (20–22). However, rs-fMRI based CVR without CO_2_ challenge (15, 16) is a relatively newer measure of CNS neurovascular function, especially in the context of HIV infection.

In addition to these neuroimaging vascular markers, plasma levels of microparticles (MPs) of endothelial and glial origin can be informative of BBB alteration. Endothelial cells (ECs) lining the inner walls of cerebral blood vessels are critical for maintenance of neurovascular homeostasis, and injury to these cells can affect many critical functions, including CBF, CVR, and BBB function. In addition, damage to endothelial cells as well as their activation can result in release of MPs by the ECs and also leakage of glial MPs into the periphery.

Microparticles are small membrane-bound micro-vesicles of 0.1–1 μM in diameter (23, 24). They act as signaling shuttles that can deliver the intracellular cargo from the cell of their origin to the recipient cell and can contribute to inflammation in various chronic conditions, including HIV infection and vascular disease, as shown by our group (25) and others (26–35). Circulating microparticles of glial origin (expressing GFAP-Glial Fibrillary acidic protein) have been considered a marker of neurovascular injury during HIV infection and other CNS disorders (36–40). Further, our group (41, 42) and others (43, 44) have shown that sonic hedgehog (Shh+) signaling during astrocyte-endothelial communication is neuroprotective and is found to be disrupted during HIV infection and can lead to increased BBB permeability. These studies indicate that MPs of glia and endothelial origins could be used as biomarkers of BBB injury. However, it is not known how these peripheral markers correlate with neurovascular neuroimaging markers.

In order to examine the effect of cART on the neurovascular function, we examined longitudinal changes in CVR, CBF, and MPs of astrocytic and endothelial origin in PWH before and after initiation of cART.

## 2. Materials and methods

This study was reviewed and approved by the Research Subjects Review Board at the University of Rochester, and all study participants signed a written informed consent prior to undergoing study procedures. Details of the study cohort have been previously reported (45). For the analyses reported here, data on 88 unique study participants, 35 (32 male) treatment-naïve PWH and 53 (27 male) age-matched HIV− healthy controls were available at baseline. However, the total number of study participants in the HIV+ cohort decreased from 35 to 32 at 12 weeks and further declined to 15 at year 2. Similarly, the total number of study participants in HIV− cohort went from 53 at baseline visit to 25 at year 2 visit. After initiation of cART the mean CD4 counts were 580 cells/μl (SD: 261) at week 12 and 848 cells/μl (SD: 271) at year 2. Similarly mean viral load was 513 copies/mL (*n* = 8, SD: 2629) at week 12 and 20 copies/mL (*n* = 10, SD: 42) at year 2. In rest of the study participants, viral loads were below detectable limit. Demographic and clinical characteristics of study participants are presented in Table 1.

**Table 1:** Demographic characteristics of study participants at baseline visit.

Characteristics	HIV+ (*N* = 35)	HIV− (*N* = 53)	*p*-value
Age, mean (SE)	34.2 (2.2)	36.9 (1.6)	0.329
**Gender, n (%)**			<0.001
Female	3 (8.6%)	26 (49.1%)	
Male	32 (91.4%)	27 (50.9%)	
**Ethnicity, n (%)**			0.383
Hispanic or Latino	3 (8.6%)	2 (3.8%)	
Not Hispanic or Latino	32 (91.4%)	51 (96.2%)	
**Race, n (%)**			<0.001
Caucasian	17 (48.6%)	43 (81.1%)	
Black.AA	18 (51.4%)	5 (9.4%)	
Other	0 (0%)	4 (7.5%)	
Missing	0 (0%)	1 (1.9%)	
**Diabetes type II, n (%)**			-
True	1 (2.9%)	1 (1.9%)	
FALSE	34 (97.1%)	48 (90.6%)	
Missing	0 (0%)	4 (7.5%)	
**High blood pressure, n (%)**			-
True	3 (8.6%)	4 (7.5%)	
FALSE	32 (91.4%)	45 (84.9%)	
Missing	0 (0%)	4 (7.5%)	
**Current smoker**			<0.001
True	16 (45.7%)	5 (9.4%)	
FALSE	19 (54.3%)	48 (90.6%)	
**Education, n (%)**			0.027
Less than or equal to 12 years	11(31.4%)	6(11.3%)	
More than 12 years	24(68.6%)	47(88.7%)	
**Previous smoker**			-
True	9 (25.7%)	14 (26.4%)	
FALSE	26 (74.3%)	39 (73.6%)	
**CD4, mean (SD), cells/uL**			
Baseline	496 (261)	-	-
CD4 Nadir	482.3 (42.7)	-	-
**Viral load, mean (SD), copies/mL**			
Baseline	90,872 (142,938)	-	-
Week 12, undetectable in *n* = 8	513 (2,629)	-	-
Year 2, undetectable in *n* = 10	20 (42)	-	-

*p*-values are obtained by either two-group Welch’s unequal variances *t*-test (for continuous variables) or Fisher exact test (for categorical variables). PWH, people with HIV; HC, HIV negative healthy controls; SE, standard error.

All participants underwent a comprehensive clinical, laboratory, and neuroimaging evaluation. PWH were assessed at baseline (BSL, prior to initiation of cART) and 12 weeks (W12), and 2 years (Y2) post-cART initiation, while healthy controls were assessed at BSL, and YR 2 only. All PWH were antiretroviral treatment-naïve prior to enrollment and met the following laboratory parameters within 30 days of baseline evaluation: hemoglobin ≥ 9.0 g/dL, serum creatinine ≤2 × ULN, AST (SGOT), ALT (SGPT), and alkaline phosphatase ≤ 2 × upper limit of normal. Participants with severe premorbid or comorbid psychiatric disorders, such as diagnoses of schizophrenia, bipolar disorder, and active depression, were excluded. Additional exclusionary criteria were stroke, head trauma resulting in the loss of consciousness for more than 30 min, multiple sclerosis, brain infections (except for HIV-1), space-occupying brain lesions requiring acute or chronic therapy, dementia as established by the HAND definition, active alcohol and drug abuse within 6 months of study entry, and conditions preventing MRI scanning (claustrophobia and metallic implants). Study participants meeting criteria for HIV-associated mild neurocognitive disorder (MND) or HIV-associated asymptomatic neurocognitive impairment (ANI) were eligible to participate. Exclusion criteria for healthy controls differ only for HIV status.

### 2.1. MRI acquisition and analysis

MRI was performed on a 3 T Siemens TIM Trio scanner (Erlangen, Germany) equipped with a 32-channel head coil at the University of Rochester’s Center for Advanced Brain Imaging and Neurophysiology (UR CABIN). A T1-weighted (T1w) 3D magnetization-prepared rapid acquisition gradient echo (MPRAGE) images were acquired with the scan parameters: inversion time (TI) = 1,100 ms, repetition time (TR)/echo time (TE) = 2,530 ms/2.33 ms, resolution = 1.0 × 1.0 × 1.0 mm^3^. Resting-state fMRI scans were acquired using a gradient echo-echo planar imaging (GE-EPI) sequence (TR/TE = 2,000 ms /30 ms,150 volumes, resolution = 4.0 × 4.0 × 4.0 mm^3^). Participants were instructed to keep their eyes closed for the duration of the sequence. Perfusion imaging was performed using a pseudo continuous arterial spin labeling (pcASL) sequence (TR/TE = 3,530/22.62 ms, resolution = 3.8 × 3.8 × 5 mm^3^) using a tag-control pair technique with 36 averages with a 1.5 s labeling duration and 1.5 s single post label delay (PLD). The equilibrium magnetization of arterial blood image (M0) was acquired with one average at a TR = 5,000 ms.

T1w images were reoriented to standard orientation, cropped, bias-field corrected, skull-stripped, linearly and nonlinearly registered to MNI152-2 mm standard space, and segmented by tissue type using FSL’s (FMRIB’s Software Library, version 5.11) anatomical processing script (fsl_anat) (46, 47). Structural images were used for co-registration of the functional and perfusion images to anatomical and standard spaces. Tissue maps were used to account for partial volume effects in functional processing. Regions of interests (ROIs) were determined *a priori* and extracted using the Harvard-Oxford cortical and subcortical atlases available in FSL. Each ROI was warped from standard to native spaces and binarized before being applied as a mask against each imaging metric map. The mean and standard deviation of each metric were calculated using FSL’s fslstats tool within each ROI. ROIs included in these analyses were the Amygdala (Amyg), Caudate Nucleus (CN), Globus Pallidus (GP), Hippocampus (Hippo), Putamen (PUT), Thalamus (TH), Precuneus Cortex (PreC), global white matter (GWM), and global gray matter (GGM). Bilateral ROIs were merged prior to calculating metric means.

The rs-fMRI data processing was carried out using FEAT (fMRI Expert Analysis Tool) Version 6.00, part of FSL (47). Registration to high-resolution structural and/or standard space images was carried out using FLIRT (48). Registration from high-resolution structural to standard space was then further refined using FNIRT nonlinear registration (49). The following pre-statistics processing was applied: motion correction using MCFLIRT (48), non-brain removal using BET (50), spatial smoothing using a Gaussian kernel of FWHM 5 mm, grand-mean intensity normalization of the entire 4D dataset by a single multiplicative factor, and high-pass temporal filtering (Gaussian-weighted least-squares straight line fitting, with sigma = 50.0 s).

CVR maps were estimated with the global signal regression coefficient method using rs-fMRI BOLD images (15), providing qualitative reactivity indices for each subject at each study visit. Briefly, the preprocessing steps encompassed motion correction, spatial smoothing through a Gaussian filter with a full-width-half-maximum (FWHM) of 4 mm, and a linear detrending. Subsequently, building upon previous findings in healthy controls that attributed global resting state BOLD fluctuations primarily to natural variations in ETCO2 levels within the frequency range of 0.02 to 0.04 Hz, we applied a band-pass filter to temporarily filter the rs-BOLD data within this specific frequency range. The gray matter tissue segmentation was then used as a mask for calculating the global average gray matter rs-fMRI signal to be used as a regressor against voxel-wise BOLD signals. The global signal has been previously shown to act as a surrogate for respiratory and cardiac fluctuations (15). The global-average gray matter rs-fMRI signal was regressed against the preprocessed BOLD signal at each voxel, where the slope of the regression was taken as a CVR index. Normalization of this index by the whole-brain average provides a qualitative estimate of CVR.

ASL images were processed with the Oxford ASL tool (51). Preprocessing included motion correction using MCFLIRT, slice-timing correction, distortion correction, spatial regularization, partial volume correction, and registration to high-resolution anatomical space using the boundary-based registration (BBR) algorithm and to 2 mm MNI-152 standard space using FNIRT nonlinear registration (52). CBF quantification was run in three steps: Bayesian inference for CBF according to the Buxton kinetic curve model, Bayesian inference of further parameters as applicable to single-post label delay data, and Bayesian inferences with spatial prior to fine tune the model parameters, initialized by the high-resolution anatomical image (53, 54).

### 2.2. Blood biomarkers

Plasma was isolated from whole blood samples from study participants by centrifugation and was cryopreserved. Plasma levels of astrocytic microparticles were determined by the expression of glial fibrillary acidic protein (GFAP) and sonic hedgehog (Shh). Endothelial-derived microparticles were assessed via expression of CD144 and CD62L. Measurements were performed by volumetric flow cytometry analysis using Accuri C6 (BD Biosciences, San Jose CA, United States) as described previously (25). Plasma levels of soluble Intracellular adhesion molecule 1 (ICAM-1) and vascular cell adhesion molecule 1 (VCAM-1) were measured by DuoSET ELISA kits from R and D systems as per manufacturer’s instructions.

### 2.3. Statistical analysis

Comparisons between two independent groups (e.g., PWH vs. control study participants at baseline) were conducted by either the two-group Welch’s unequal variances *t*-test (for continuous variables) or Fisher exact test (for categorical variables). Paired *t*-tests were used to compare the levels of continuous variables in PWH study participants between time points baseline, 12 week, and year two visits. A value of *p* < 0.05 was considered statistically significant for a single hypothesis testing problem. In the subset of subjects with blood biomarkers, we examined the relationships between these markers and regional CVR and CBF metrics. We performed the Spearman Correlation Test between the blood markers and regional imaging metrics at each study visit. The Benjamini-Hochberg multiple testing procedure was utilized to regulate the false discovery rate (FDR) at a significant level of 0.05. All statistical analyses were performed in R 3.6.2 (R Foundation for Statistical Computing, Vienna, Austria) and GraphPad Prism (version 9.0, GraphPad Software, Inc., San Diego, CA).

## 3. Results

### 3.1. HIV, cART, and neuroimaging markers

**Figure 1:**
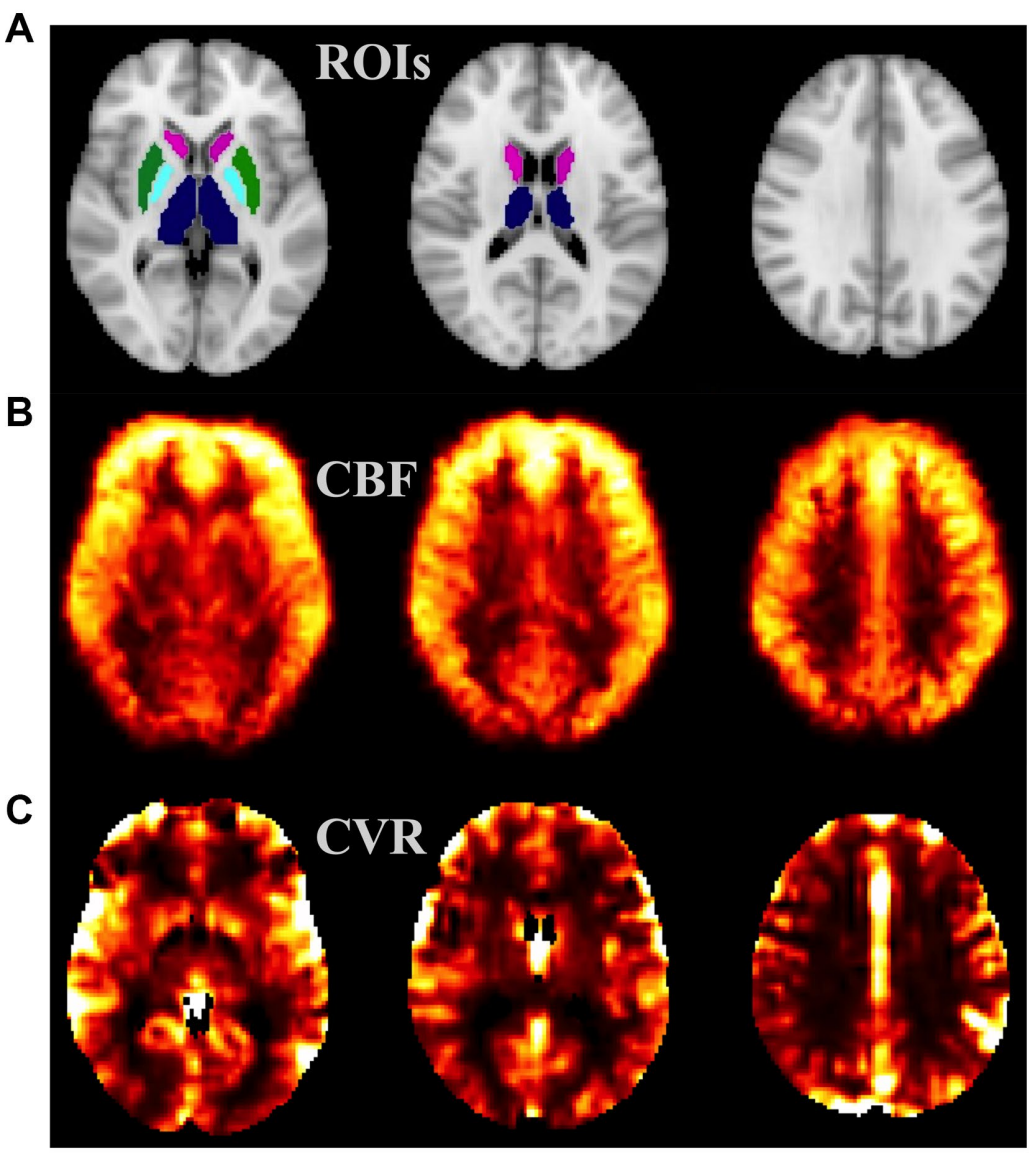
Example MRI maps from a healthy subject: **(A)** ROIs considered—TH (blue), CN (magenta), PUT (green), and GP (cyan); **(B)** cerebral blood flow (CBF) map, intensity scale: 0–50 mL/100 g/min; **(C)** cerebrovascular reactivity (CVR) map, intensity scale: 0–3 relative unit. CN, caudate nucleus; PUT, putamen; GP, Globus Pallidus; ROI, region of interest.

**Figure 2:**
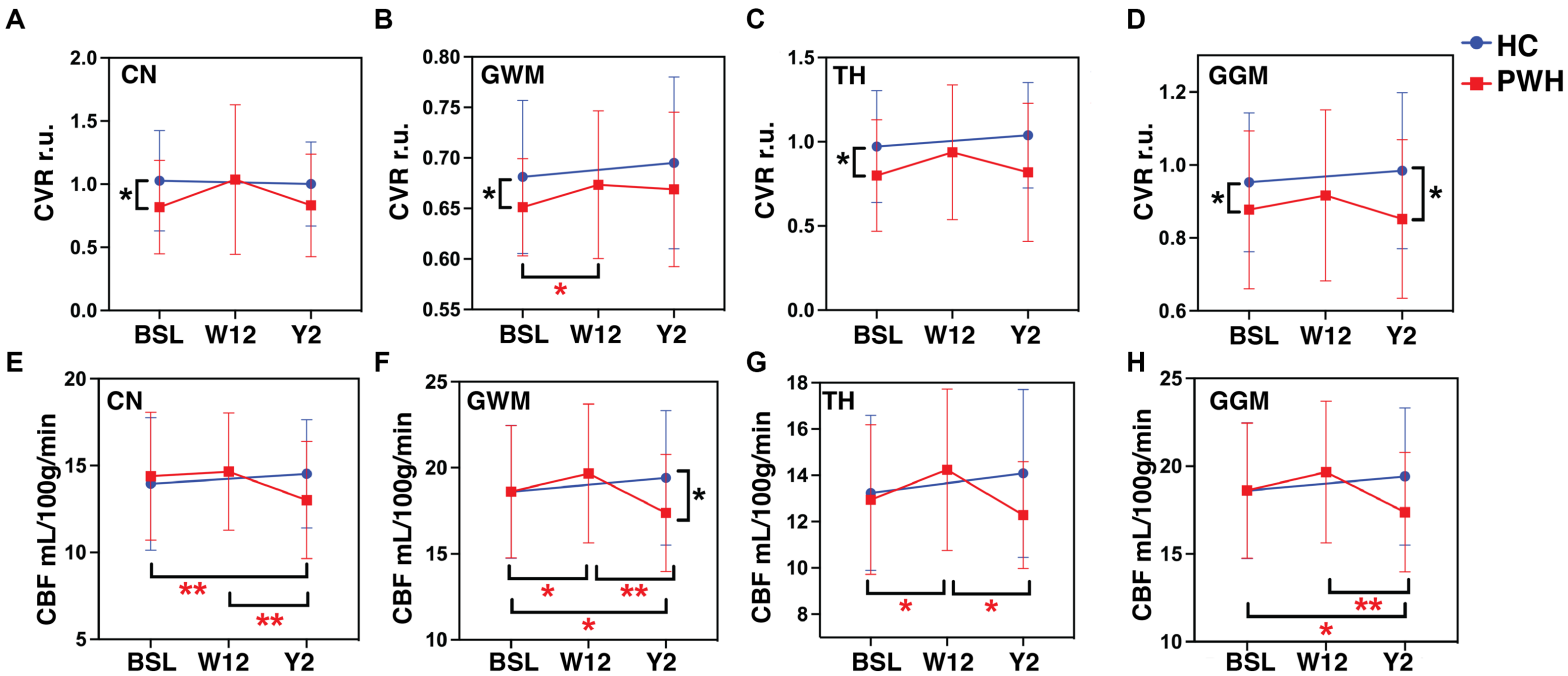
Neuroimaging markers. The plots represent CVR in **(A)** CN, **(B)** GWM, **(C)** TH, **(D)** GGM and CBF in **(E)** CN, **(F)** GWM, **(G)** TH, and **(H)** GGM at baseline, week-12 and year-2 for PWH cohort in red and baseline and year 2 for HC cohort in blue color. ^*^p < 0.05, ^**^p < 0.01 by paired *t*-tests. CVR, cerebrovascular reactivity; CBF, cerebral blood flow; r.u., relative unit.

Figure 1 presents CVR and CBF maps from a study participant with HIV infection. Among all the different ROIs measured, only CN, TH, GWM, and GGM exhibited statistically significant differences between groups; hence, results pertaining to only these three ROIs are discussed. Univariate analysis showed that at baseline, CVR was significantly lower in CN (*p* = 0.015), TH (*p* = 0.022), GWM (*p* = 0.018) and GGM (*p* = 0.02) in PWH as compared to HIV uninfected controls (Figures 2A–D). During longitudinal follow-up, at 2 years, in HIV− controls, CVR values remain unchanged. In PWH, after initiation of cART, there was a moderate increase in CVR in all three regions (statistically significant only in GWM, *p* = 0.042) at 12 weeks post-treatment, suggesting a beneficial effect of the treatment. At 2 years post-treatment, however, CVR values show a decreasing trend, albeit not to the same levels as at baseline, with statistical significance only in GGM (*p* = 0.02, Figure 2D). Univariate analysis showed no difference in CBF (measured as mL/100 grams of brain tissue/min) at baseline between PWH and control groups (Figures 2E–H). Similar to CVR, CBF remains almost unchanged in longitudinal follow-up in healthy controls. In PWH there was an increase in CBF at week 12 (statistically significant in GWM, *p* = 0.039 and TH, *p* = 0.01), followed by a decline at 2 years of follow-up (statistically significant in GWM, *p* = 0.017 and GGM, *p* = 0.023, as compared to baseline visit). Interestingly, there was a significant decline in CBF at year 2 as compared to week 12 time point in all four ROIs, CN, GWM, TH, and GGM (*p* = 0.0017, 0.0024, 0.0122, and 0.0018, respectively). This trend is similar to what we observed for CVR.

### 3.2. HIV, cART, and blood biomarkers

**Figure 3:**
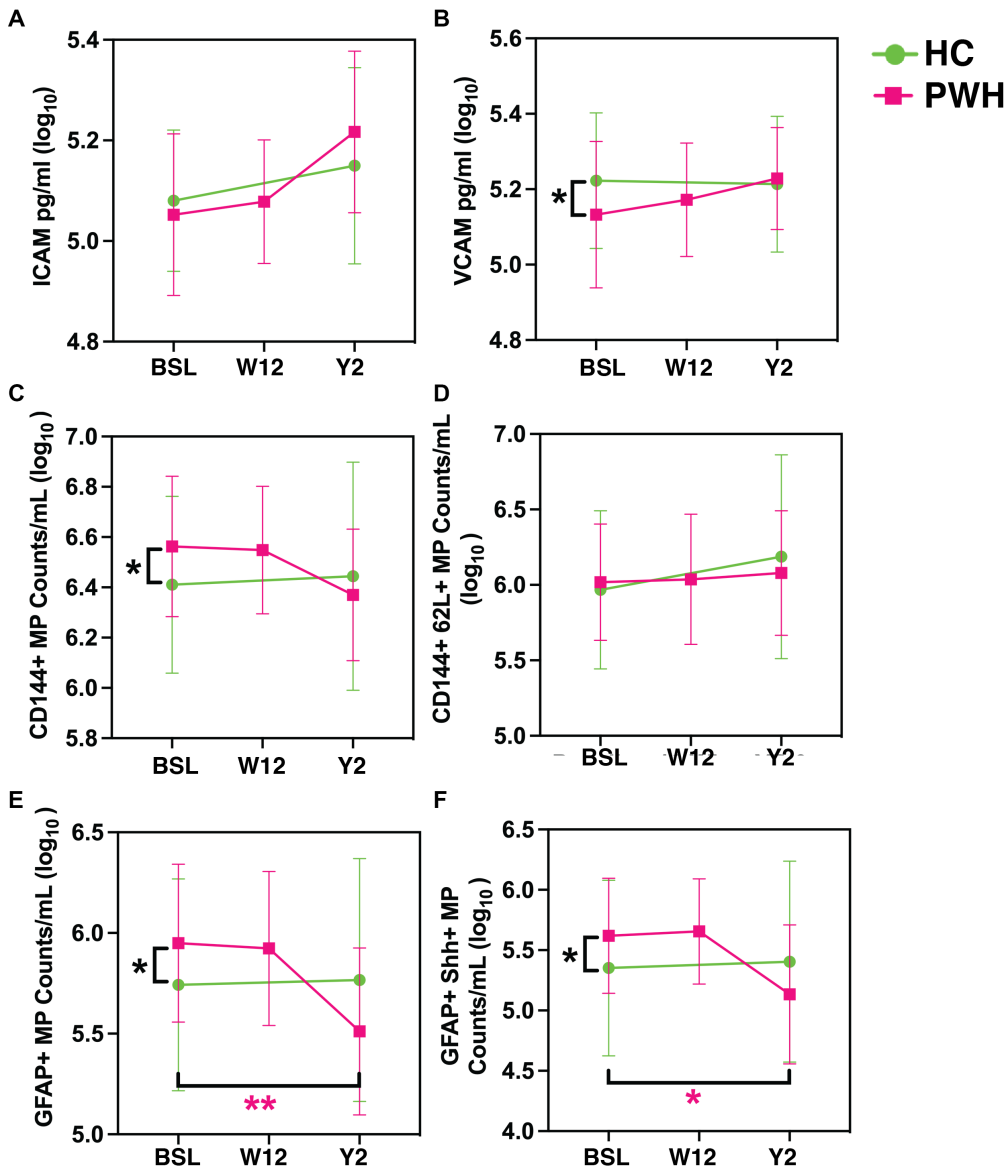
Blood biomarkers. The plots represent plasma levels of **(A)** ICAM-1, **(B)** VCAM-1, **(C)** CD144+ endothelial MPs, **(D)** CD144+CD62L+ endothelial MPs, **(E)** GFAP+ astrocytic MPs, and **(F)** GFAP+Shh+ astrocytic MPs at baseline, week-12 and year-2 for HIV+ cohort in red and baseline and year 2 for HIV− cohort in green. ^*^p < 0.05, ^**^p < 0.01, ^***^p < 0.001 by paired *t*-tests. MPs, microparticles.

Univariate analysis did not show any significant difference in plasma levels of ICAM-1 between the two groups at any time point. There was a trend toward increase in ICAM-1 levels within both the groups, at each successive visit but the difference was not statistically significant (Figure 3A).

With respect to VCAM, at baseline, HIV uninfected group had higher plasma levels compared to PWH (Figure 3B; *p* = 0.0427). This difference did not persist at the 2-year follow-up. In addition to these well-known markers of endothelial activation, we also measured microparticles of endothelial (CD144+ MPs, CD144+CD62L+ MPs) and glial (GFAP+ MPs, GFAP+Shh+ MPs) origin. With the exception of CD144+CD62L+ MPs, all other types showed significantly higher levels in PWH compared to controls at baseline (Figures 3C–F; *p* = 0.016, 0.0239, 0.0092, respectively). The differences between the two groups were not statistically different after 2 years of cART treatment. Within the PWH group, the largest treatment effect was observed in GFAP+ MPs and GFAP+Shh+ MPs.

### 3.3. Correlations between neuroimaging and blood biomarkers

**Figure 4:**
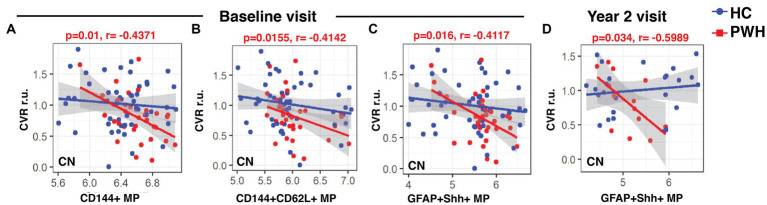
Associations between CVR and blood biomarkers. The linear regression plots show CVR in CN at baseline in PWH cohort showed significant negative correlations with **(A)** CD144+ and **(B)** CD144+CD62L+ endothelial MPs and also with **(C)** GFAP+Shh+ astrocytic MPs. **(D)** CVR at year-2 continued to show string negative correlations with CVR in PWH cohort. CVR, cerebrovascular reactivity; r.u., relative unit.

At the baseline visit, CVR in CN showed significantly negative correlations with CD144+ MPs (r: −0.4371, *p* = 0.01), CD144+CD62L+ MPs (r: −0.4142, *p* = 0.0155) and GFAP+Shh+ MPs (r: −0.4117, *p* = 0.016) only in PWH (Figures 4A–C, respectively). While there were no correlations at the week 12 visit, GFAP+Shh+ MPs were again significantly negatively correlated with CVR in CN at year 2 in PWH (Figure 4D, r: −0.5989, *p* = 0.034). CVR in any regions did not correlate with any blood markers in controls at any time point (data not shown).

**Figure 5:**
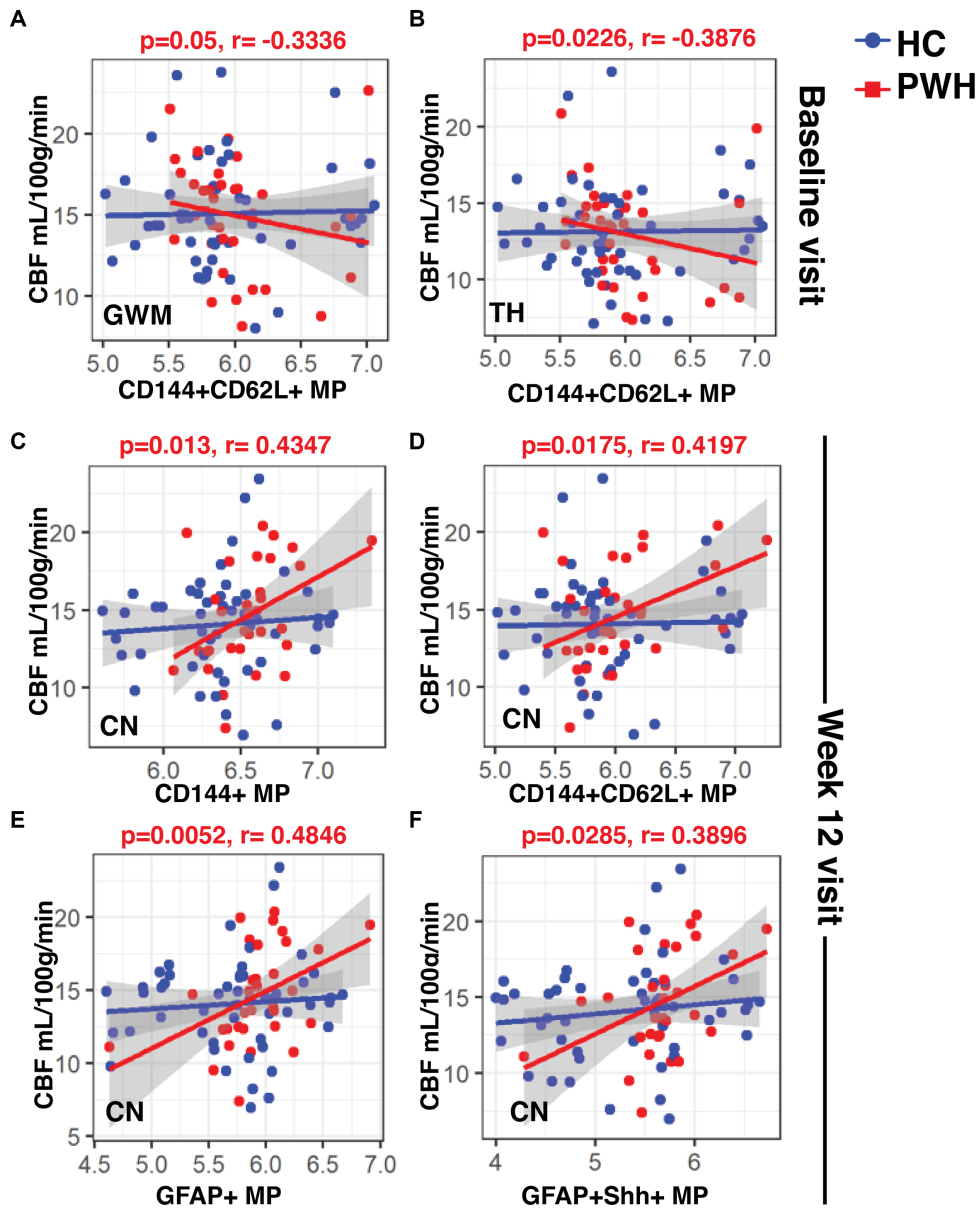
Associations between CBF and blood biomarkers. The linear regression plots show **(A)** CBF in GWM and **(B)** TH at baseline in PWH cohort showed significant negative correlations with CD144+CD62L+ endothelial MPs. **(C–F)** CBF CN in PWH cohort at week-12 correlated positively with all types of MPs. CBF, cerebral blood flow; MPs, microparticles.

In the case of CBF, at baseline, the only significant negative correlations were between CD144+CD62L MPs and CBF in GWM (Figure 5A; r: −0.3336, *p* = 0.05) and CBF in TH (Figure 5B; r: −0.3876, *p* = 0.0226) in the PWH. Interestingly, at week 12, all four types of MPs (CD144+, CD144+CD62L+, GFAP+ and GFAP+Shh+) correlated positively with CBF in CN (Figures 5C–F; r: 0.4347, *p* = 0.013; r: 0.4197, *p* = 0.0175; r: 0.4846, *p* = 0.0052; r: 0.3896, *p* = 0.0285, respectively) but there were no correlations at year 2 (data not shown). Similar to CVR, controls did not show any correlations between CBF and blood biomarkers.

## 4. Discussion

The results from this study suggest that CVR, endothelial, and glia MPs are early markers of neurovascular dysfunction in cART naïve PWH. Most of these markers improved shortly after cART treatment; however, the beneficial effect of cART treatment diminished over time. Abnormalities in CVR were found in both subcortical gray matter regions (CN and TH) and white global white matter (GWM), indicating a diffuse involvement of the brain microcirculation. The trend of decreased CVR during chronic cART-treated PWH was associated with a decrease in CBF, especially in the GWM.

Two recent studies have also assessed CVR in PWH using alternative approaches to ours. Callen et al. (55) used changes in CBF induced by intravenous acetazolamide and found decreased CVR in the frontal lobe and basal ganglia. Chow et al. (56) used transcranial Doppler ultrasound (TCD) and breath-holding to assess mean flow velocity changes to infer CVR. Higher CVR was associated with a higher score on the Montreal Cognitive Assessment.

Since the cohort of PWH we enrolled was relatively young (average age 34 years) with preserved immune function before starting cART (average nadir and baseline CD4+ cell count > 400 cell/uL), it could be envisioned that PWH as they age would be further exposed to CBVD and in particular to cerebral small vessel disease (CSVD). CVR is closely linked to the regulation of the microcirculation, thus, is reduced in CSVD (57). Consistent with these implications are recent studies showing a higher prevalence of CSVD in PWH compared to HC (2, 58).

CBF in our study did not differ between PWH cART naïve prior to starting treatment and controls, suggesting that more significant alteration in tissue perfusion is necessary before changes in CBF can be captured. In contrast, CVR is more closely related to CSVD (59).

CVR is a marker of endothelial function; therefore, we assessed the relationship between CVR and plasma levels of endothelial-derived MPs. We measured MPs that expressed endothelial marker CD144 (CD144+ MPs) and also MPs double positive for CD144 and CD62L (aka L-selectin), which is a marker of endothelial activation (CD144+CD62L+ MPs). Endothelial markers, including ICAM-1 and VCAM-1 were also measured. The increased plasma levels of CD144+ MPs in PWH compared to controls are consistent with the expected effect of inflammation before cART initiation and the subsequent decline with reduced inflammation during cART. In contrast, VCAM-1 level before cART were surprisingly lower in PWH and normalized with treatment. This finding needs to be confirmed in further investigations. On the other hand, ICAM-1 did not differ between groups.

Systemic inflammation associated with HIV infection could also lead to an altered BBB. In this study, we observed that GFAP+ MPs were elevated in cART naïve PWH, suggesting an increased BBB permeability. The beneficial response to cART treatment continued during the two-year follow-up. We next examined the temporal correlation between CVR and blood biomarkers. CVR in CN correlated negatively with endothelial and glial MP levels at baseline study visit, only in PWH. At year-2, despite a smaller number of study participants, glial MPs continue to show an even stronger negative correlation with CVR in caudate. Endothelial MPs also correlated negatively with CBF in GWM and Thalamus at baseline visit in PWH. However, after cART both MPs and CBF decreased, making now their relationship positively correlated but likely not causally related. From these results, CVR and endothelial and glia MPs provide a consistent mechanistic relationship when assessing cerebral microcirculation. When assessing CVR directly is not possible, for example in resource-limited settings where MRI facilities are not readily available, these blood biomarkers could be more easily implemented.

Our study has a few limitations. First, the sample size of our study is small due to both enrollment restrictions/difficulties with a cART-naïve population and a high attrition rate. Second, as the study recruited study participants that were treatment-naïve to cART, a young study population limited the evaluation of the effects of cART in older PWH. Also, PWH in this geographical location are heavily skewed toward male sex-at-birth. Owing to that, we were able to enroll only three cART naïve female study participants and thus were not able to evaluate the effect of sex. Furthermore, the calculation of CVR from resting-state fMRI has limitations as well. While methods have been developed to qualitatively assess a relative CVR index based on resting-state data (15, 16), the gold standard to quantify and assess CVR is using a vasoactive stimulus task-based breathing challenge (60). An additional limitation is the post label delay (PLD) of 1.5 s used in ASL, shorter than the 1.8 s currently recommended (61). It should be noted that our protocol was implemented prior to this recommendation. However, the relative young age of the cohort enrolled likely minimizes the impact of the shorter PLD. Despite these caveats, our study design allows for a unique approach to study and separate the effects of acute and chronic HIV infection and cART treatment on brain cerebrovascular function.

## 5. Conclusion

To the best of our knowledge, this is the only study that specifically investigates longitudinal changes in CVR, CBF, endothelial, and glia MPs before and after cART initiation. Our findings indicate that untreated HIV infection, even with relatively preserved immune function, affects brain microcirculation. While cART normalizes endothelial and glia MPs levels, effects on CVR and CBF seem less effective over time, possibly predisposing PWH to CSVD.

## Data availability statement

The original contributions presented in the study are included in the article/supplementary material, further inquiries can be directed to the corresponding author.

## Ethics statement

The studies involving humans were approved by University of Rochester Research Subject Review Board. The studies were conducted in accordance with the local legislation and institutional requirements. The participants provided their written informed consent to participate in this study.

## Author contributions

MS and MU: study design, conducting experiments, acquiring data, analyzing data, and writing the manuscript. VS, AP, and YZ: conducting experiments, acquiring data, and analyzing data. KM: conducting experiments, acquiring data, analyzing data, and writing the manuscript. AT: data entry and database management. LW: statistical support. MT and JZ: manuscript review for intellectual content. XQ: statistical support and manuscript review for intellectual content. GS: study design, funding acquisition, and manuscript writing and review for intellectual content. All authors contributed to the article and approved the submitted version.

## Funding

This work was supported by the National Institutes of Health, grant numbers R01MH099921 and R01AG054328.

## Conflict of interest

The authors declare that the research was conducted in the absence of any commercial or financial relationships that could be construed as a potential conflict of interest.

## Publisher’s note

All claims expressed in this article are solely those of the authors and do not necessarily represent those of their affiliated organizations, or those of the publisher, the editors and the reviewers. Any product that may be evaluated in this article, or claim that may be made by its manufacturer, is not guaranteed or endorsed by the publisher.

## Figures and Tables

**Table:** 

cART	Combination antiretroviral therapy
PWH	People with HIV
HC	HIV negative healthy controls
CBF	Cerebral blood flow
CVR	Cerebrovascular reactivity
CBVD	Cerebrovascular disease
CSVD	Cerebral small vessel disease
CNS	Central nervous system
ASL	Arterial spin labeling
rs-fMRI	Resting-state functional magnetic resonance imaging
GFAP	Glial fibrillary acidic protein
BBB	Blood brain barrier
Shh	Sonic hedgehog
BSL	Baseline
W12	Week 12
Y2	Year 2
MND	Mild neurocognitive disorder
ANI	Asymptomatic neurocognitive impairment
MPRAGE	Magnetization-prepared rapid acquisition gradient echo
TR	Repetition time
TE	Echo time
pCASL	Pseudo continuous arterial spin labeling
ROI	Regions of interest
Amyg	Amygdala
CN	Caudate nucleus
GP	Globus Pallidus
Hippo	Hippocampus
PUT	Putamen
TH	Thalamus
PreC	Precuneus Cortex
GWM	Global white matter
BOLD	Blood oxygen dependent
BBR	Boundary-based registration
ICAM-1	Intracellular adhesion molecule 1
VCAM-1	Vascular cell adhesion molecule 1
MPs	Microparticles
SE	Standard error
PLD	Post label delay
M0	Equilibrium magnetization of arterial blood image
